# Emergence of T cell immunosenescence in diabetic chronic kidney disease

**DOI:** 10.1186/s12979-020-00200-1

**Published:** 2020-10-20

**Authors:** Yen-Ling Chiu, Wan-Chuan Tsai, Ruo-Wei Hung, I-Yu Chen, Kai-Hsiang Shu, Szu-Yu Pan, Feng-Jung Yang, Te-Tien Ting, Ju-Ying Jiang, Yu-Sen Peng, Yi-Fang Chuang

**Affiliations:** 1 Graduate Program in Biomedical Informatics, Department of Computer Science and Engineering, College of Informatics, Yuan Ze University, Taoyuan, Taiwan; 2 Division of Nephrology, Department of Medicine, Far Eastern Memorial Hospital, New Taipei City, Taiwan; 3 Graduate Institute of Clinical Medicine, National Taiwan University College of Medicine, Taipei, Taiwan; 4 Center for General Education, Lee-Ming Institute of Technology, New Taipei City, Taiwan; 5 Graduate Institute of Immunology, National Taiwan University College of Medicine, Taipei, Taiwan; 6 Department of Medicine, National Taiwan University Hospital Yun Lin Branch, Douliu, Taiwan; 7 School of Big Data Management, Soochow University, Taipei, Taiwan; 8 Division of Endocrinology and Metabolism, Department of Medicine, Far Eastern Memorial Hospital, New Taipei City, Taiwan; 9 Department of Applied Cosmetology, Lee-Ming Institute of Technology, New Taipei City, Taiwan; 10 Department of Healthcare Administration, Oriental Institute of Technology, New Taipei City, Taiwan; 11 Institute of Public Health, School of Medicine, National Yang-Ming University, Taipei, Taiwan

**Keywords:** Immunosenescence, CKD, T cell, Diabetes, BMI

## Abstract

**Background:**

Type 2 diabetes is an important challenge given the worldwide epidemic and is the most important cause of end-stage renal disease (ESRD) in developed countries. It is known that patients with ESRD and advanced renal failure suffer from immunosenescence and premature T cell aging, but whether such changes develop in patients with less severe chronic kidney disease (CKD) is unclear.

**Method:**

523 adult patients with type 2 diabetes were recruited for this study. Demographic data and clinical information were obtained from medical chart review. Immunosenescence, or aging of the immune system was assessed by staining freshly-obtained peripheral blood with immunophenotyping panels and analyzing cells using multicolor flow cytometry.

**Result:**

Consistent with previously observed in the general population, both T and monocyte immunosenescence in diabetic patients positively correlate with age. When compared to diabetic patients with preserved renal function (estimated glomerular filtration rate > 60 ml/min), patients with impaired renal function exhibit a significant decrease of total CD3^+^ and CD4^+^ T cells, but not CD8^+^ T cell and monocyte numbers. Immunosenescence was observed in patients with CKD stage 3 and in patients with more severe renal failure, especially of CD8^+^ T cells. However, immunosenescence was not associated with level of proteinuria level or glucose control. In age, sex and glucose level-adjusted regression models, stage 3 CKD patients exhibited significantly elevated percentages of CD28^−^, CD127^−^, and CD57^+^ cells among CD8^+^ T cells when compared to patients with preserved renal function. In contrast, no change was detected in monocyte subpopulations as renal function declined. In addition, higher body mass index (BMI) is associated with enhanced immunosenescence irrespective of CKD status.

**Conclusion:**

The extent of immunosenescence is not significantly associated with proteinuria or glucose control in type 2 diabetic patients. T cells, especially the CD8^+^ subsets, exhibit aggravated characteristics of immunosenescence during renal function decline as early as stage 3 CKD. In addition, inflammation increases since stage 3 CKD and higher BMI drives the accumulation of CD8^+^CD57^+^ T cells. Our study indicates that therapeutic approaches such as weight loss may be used to prevent the emergence of immunosenescence in diabetes before stage 3 CKD.

## Introduction

Diabetic kidney disease is a growing, worldwide public health epidemic. There are more than 415 million people suffering from diabetes worldwide, and it is estimated that more than 40% of these patients will develop chronic kidney disease (CKD), creating a huge health care burden [1, 2]. Beyond the burden of care for renal failure itself, diabetic kidney disease increases the risk for other comorbidities, especially cardiovascular disease (CVD) [3] and infection [4]. Unfortunately, despite much progress in the treatment of hypertension and hyperlipidemia to reduce CVD, the prevalence of CVD in diabetic patients with renal failure remains excessively high [5]. Patients with type 2 diabetes are also at higher risk for infections of the skin and soft tissue, genitourinary, gastrointestinal and respiratory tracts [6], and are twice as likely to be hospitalized for infectious disease management than non-diabetics. Overall, the prevalence of diabetics and diabetic complications is expected to continue rising due to the increasing predominance of obesity and advanced age in the human population.

Immune mechanisms are increasingly being recognized as important factors in the development of various aging-associated chronic conditions [7, 8]. During normal aging, the continuous interaction between the host and environment results in accumulating changes in the immune system, which is collectively referred to as *immunosenescence* [9, 10]. This complex process of immune aging leads to increases in specific changes in T cell and monocyte populations and is found to be significantly enhanced in individuals with chronic diseases associated with inflammation [11], such as end-stage renal disease [12, 13] and rheumatoid arthritis [14].

As the host encounters antigens, there is a progressive decrease in naïve T cells and concomitant increase in memory T cells of the effector phenotype [15]. Over time, T cells gradually lose the expression of CD28 and CD27 costimulatory molecules as well as the hemostatic cytokine receptor CD127 from their surface. The loss of naïve T cells leads to decreased vaccine responses and poor control of newly encountered pathogens [16]. On the other hand, the accumulation of memory T cells is highly proinflammatory and these cells acquire the expression of senescent cell markers such as CD57 and KLRG1. Moreover, these advanced effector T cells with increased production of proinflammatory cytokines and adhesion molecules are more easily recruited to the vascular epithelium, which may lead to the development of atherosclerotic CVD [17, 18].

In addition to T lymphocytes, monocytes in the peripheral blood also exhibit changes during aging. In humans, circulatory CD68^+^ monocytes can be separated into three major subsets based on their CD14 and CD16 expression. The percentage of proinflammatory CD14^+^CD16^+^ monocytes reportedly increases with age and correlates with cardiovascular mortality [19, 20]. We recently developed a comprehensive aging-related immune profile based on both lymphocytes and monocytes and demonstrated that aggravated immunosenescence is positively associated with CVD in end-stage renal disease (ESRD) patients [21]. Furthermore, a collective aging-related immune matrix derived from a 9-year longitudinal study of innate and adaptive immunity markers was found to be predictive of all-cause mortality beyond the well-established risk factors listed in the Framingham Heart Study [22]. Thus, immunosenescence is an important phenomenon of aging that requires further understanding including of the development of aging-related diseases and cardiovascular complications. In this manuscript, we investigated whether patients with type 2 diabetes exhibit significant changes related to immunosenescence with age and if these changes develop as renal function declines.

## Methods

### Participants

All study participants were recruited from the Far Eastern Memorial Hospital outpatient clinic between January and December of 2018. Only individuals diagnosed with type 2 diabetes for more than 6 months and older than 20 years were enrolled. The average age was 62 ± 10.3 years and mean duration of diagnoses of diabetes was 10 years. Patients who had been hospitalized in the 3 months prior to the study or who were diagnosed with active cancer were excluded. The study protocol was proved by the institutional review board of the Far-Eastern Memorial Hospital (FEMH 107032-F).

### Data collection and laboratory exams

Clinical data were collected by trained study assistants and peripheral blood was collected on the day of informed consent. Through history taking and detailed chart reviews, baseline co-morbidities and clinical laboratory data were recorded. All clinical laboratory exams including high sensitivity C-reactive protein (hs-CRP) nephelometry (Siemens) were tested in the core laboratory of the Far Eastern Memorial Hospital on the day of blood collection. Renal function was assessed using eGFR (estimated glomerular filtration rate), which was calculated using the CKD-EPI formula based on serum creatinine level. Patients were separated into three different groups: patients with normal eGFR > 60 ml/min (*n* = 379), patients with stage 3 CKD (*n* = 110, eGFR between 30 and 60 ml/min), and patients with stage 4 or stage 5 CKD (*n* = 34, eGFR less than 30 ml/min).

### Peripheral blood immunophenotyping

Immediately after blood collection, 100 μl of whole blood was immediately stained with the lymphocyte or monocyte panel and results were acquired using a Beckman Coulter CytoFLEX multicolor flow cytometer on the same day. For the lymphocyte panel, CD45-KrO (clone J33, Beckman Coulter) was used to identify leukocytes and CD3-AF700 (clone UCHT1, Biolegend) was used to identify CD3^+^ T cells from the lymphocytes gated by forward and side scatter properties. CD4^+^ and CD8^+^ T cells were stained with CD4-BUV405 and CD8-APC/Cy7 antibodies (clone RPA-T4, BD and SK1, Biolegend). T cell differentiation states were determined by staining with CD27-PE/Cy7 and CD45RO-Alexa488 antibodies (clone O323 and UCHL1, Biolegend). Additional phenotypic markers were identified with CD28-BV421 (clone CD28.2, Biolegend), CD57-APC (clone HCD57, Biolegend), and CD127-PE (clone eBioRDR5, eBioscience) antibodies.

After gating on forward/side scatter and CD45, as in the lymphocyte panel, total monocytes were first identified by staining with CD86-PE antibody (clone IT2.2, eBioscience), and were further classified as classical (CD14^++^CD16^−^), intermediate (CD14^++^CD16^+^), and non-classical (CD14^+^CD16^++^) subsets by staining with CD14-FITC and CD16-APC/Cy7 antibodies (clone M5E2 and clone 3G8, both from Biolegend).

Absolute cell number counting based on the fluidics of the cytometer was performed according to manufacturer’s protocol (CytoFLEX, Beckman Coulter) following the quality assurance procedure.

### Statistical analyses

Patient characteristics and immunophenotyping results are described as mean ± standard deviation for continuous variables and frequency for categorical variables. These variables were analyzed by analysis of variance (ANOVA) and Chi-square test, respectively, when compared between groups of different renal function. Pearson correlation was applied to evaluate the correlation of age with immune cell subset frequencies and absolute cell numbers. Multivariate linear regression models with immunophenotyping results as the dependent variable, adjusting for age and gender, were used to investigate the associations between renal function and immunophenotyping results. Trend analysis was also conducted to investigate the linear trend between immunophenotyping and stages of CKD. All statistical tests were two-tailed, and a *p* value of less than 0.05 was considered significant. The statistical analyses were performed with STATA version 15.1 (StataCorp).

## Results

### Age-related immune changes identified in type 2 diabetic patients

**Fig. 1:**
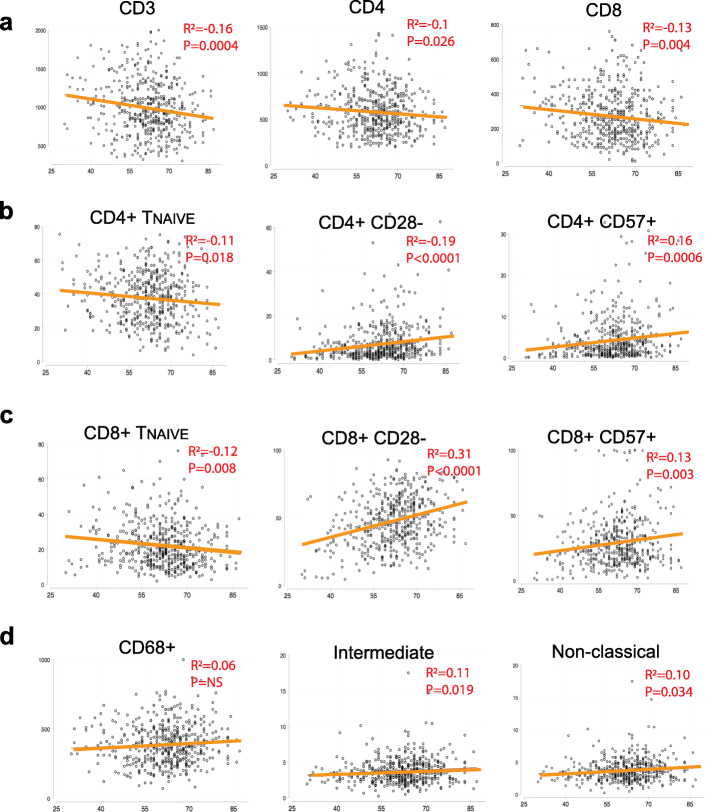
Correlations between immune cell number and subset with age. Bivariate Pearson’s correlation plots between immune cell number (CD3+, CD4+, CD8+ T cells and CD68+ total monocytes), immune cell percentage (all the others) with age. Refer to supplementary Table 1 for the complete analysis result. In Fig. 1, all the *p* values for the plots here are statistically significant (< 0.05) with the exception of the CD68+ total monocyte cell number

We first analyzed the relationship between age and immune cell numbers as well as frequencies. Supplementary Figure 1 shows a representative multicolor flow cytometry experiment. As shown in Fig. 1a and supplementary Table 1, a progressive decrease in total CD3^+^ T cell numbers as well as CD4^+^ and CD8^+^ T cells were noted with advancing age. However, the total number of CD68^+^ monocytes did not change with age (Fig. 1d). T cell differentiation was determined by the pattern of CD27 and CD45RO expression as well as through analysis of additional markers associated with aging (CD127, CD27, CD28 and CD57). The classification of progressive T cell differentiation status based on CD27 and CD45RO is as follows: Naïve T cells (T_NAIVE_): CD27^+^CD45RO^−^; central memory T_CM_ cells: CD27^+^CD45RO^+^; effector memory T_EM_ cells: CD27^−^CD45RO^+^; and the most differentiated effector T_E_ cell subset: CD27^−^CD45RO^−^. When the subset composition of CD4^+^ and CD8^+^ T cells (Fig. 1b and c) was analyzed, there was a significant progressive loss of naïve T cells and increase of memory cells (effector memory T_EM_ and effector T_E_ subsets) with age, which is consistent with reported findings in healthy individuals. Furthermore, the homeostatic CD127^+^ (IL-7 receptor) subset was found to decrease with age, while CD28^−^, CD27^−^, and CD57^+^ senescent cell subsets increased with age. Proinflammatory CD14^++^CD16^+^ and non-classical CD14^+^CD16^++^ monocytes also increased in frequency with age, albeit to a much lesser extent than the changes seen in lymphocytes. These findings are compatible with the general immunosenescence changes associated with aging.

### Patient characteristics in different estimated glomerular filtration rate (eGFR) groups

To understand if patients with diabetic kidney disease exhibit more immunosenescence features than patients with normal renal function, participants were separated into three groups: patients with normal eGFR > 60 ml/min, patients with stage 3 CKD (eGFR between 30 and 60 ml/min), and patients with stage 4 or stage 5 CKD (eGFR less than 30 ml/min). Clinical characteristics of the three groups including their glucose control and medications were compared and are shown in Table 1. Patients with impaired renal function were older than patients with preserved renal function. As expected, patients with stage 4/5 CKD had an average eGFR of 19.6 ml/min and decreased serum albumin levels compared to the other two groups. Blood sugar was slightly higher for patients with stage 3 CKD than other groups, as was systemic inflammation, as measured by hs-CRP. Furthermore, there was a higher percentage of insulin use and lower percentage of sulfonylurea usage among patients with stage 4/5 CKD.

**Table 1:** Baseline demographics, clinical, and laboratory measurements stratified by CKD stage based on eGFR

	eGFR ≥60 (*n* = 379)	Stage 3 CKD (*n* = 110)	Stage 4/5 CKD (*n* = 34)	*p* value
eGFR (ml/min)	88.3 (15.4)	48.0 (8.0)	19.7 (7.4)	< 0.001*
Age (yr)	59.9 (10.1)	67.7 (8.6)	67.1 (8.2)	< 0.001*
Male (%)	197 (52.0)	61 (55.5)	26 (76.5)	0.022
Systolic BP (mmHg)	130.5 (16.5)	135.4 (16.8)	132.8 (13.5)	0.019
Diastolic BP (mmHg)	71.9 (11.6)	70.4 (12.8)	70.4 (11.2)	0.44
Albumin (g/dl)	4.5 (0.3)	4.4 (0.3)	4.1 (0.5)	< 0.001*
Creatinine (mg/dl)	0.8 (0.2)	1.4 (0.3)	3.6 (2.0)	< 0.001*
HbA1c (%)	7.2 (1.0)	7.3 (1.1)	7.1 (1.0)	0.41
AC sugar (mg/dl)	137.7 (37.4)	145.9 (61.6)	124.7 (31.6)	0.034*
Use of insulin (%)	93 (24.5)	49 (44.5)	22 (64.7)	< 0.001*
Use of sulfonylurea (%)	167 (44.1)	46 (41.8)	8 (23.5)	0.067
Usage of metformin (%)	320 (84.4)	49 (44.5)	**	< 0.001
Use of DPP4i (%)	164 (43.3)	51 (46.4)	13 (38.2)	0.69
Use of SGLT2i (%)	43 (11.3)	**	0 (0.0)	< 0.001
T-Cholesterol (mg/dl)	145.1 (30.8)	143.6 (26.2)	141.0 (25.3)	0.70
LDL (mg/dl)	87.4 (25.1)	83.8 (26.0)	82.4 (20.8)	0.27
Triglyceride (mg/dl)	136.6 (109.4)	162.8 (103.0)	157.7 (104.7)	0.060
hs-CRP (mg/dl)	0.21 (0.32)	0.47 (1.12)	0.33 (0.61)	< 0.001*

Demographic and clinic data were compared between eGFR groups. Continuous variables are described as mean (SD) for parametric variables and median (interquartile range) for nonparametric variables, unless otherwise indicated. The *P* values were calculated using one-way ANOVA for continuous variables and X^2^ tests for categorical variables. Values are expressed as means (SD). *: *p* value < 0.05. eGFR, estimated glomerular filtration rate derived from the CKD-EPI formula. Stage 3 CKD, eGFR between 30 and 60; stage 4 CKD, eGFR between 15 and 30; stage 5 CKD, eGFR less than 15. DPP4i: Dipeptidyl peptidase-4 inhibitor. SGLT2i: sodium-glucose cotransporter 2 inhibitor. **cell number < 5

### Relationship between CKD status and immunosenescence

We next examined the immunosenescence profile of each patient group (Table 2). Total CD3^+^ and CD4^+^ T cell numbers decreased with more severe CKD, reminiscent of aging and patients on hemodialysis [21]. Although no decrease in naïve CD4^+^ or CD8^+^ T cells was found, there was a significant decrease of CD127^+^ T cells in both the CD4^+^ and CD8^+^ compartments. CD8^+^ effector cells, both CD28^−^ and CD57^+^ subsets, significantly increased in the two CKD groups compared to individuals with normal eGFR. Unexpectedly, no changes in monocyte subset distribution were detected between groups.

**Table 2:** Immune cell number and phenotype comparisons by CKD stage

	eGFR ≥60 (*n* = 379)	Stage 3 CKD (*n* = 110)	Stage 4/5 CKD (*n* = 34)	*p* value
Number CD3+	1023.2 (418.1)	964.8 (447.5)	788.0 (311.2)	0.005*
Number CD4+	604.4 (277.0)	555.3 (248.7)	450.1 (200.7)	0.003*
Number CD8+	282.8 (158.6)	293.1 (190.4)	221.4 (134.0)	0.079
CD4+ T_NAIVE_ (%)	37.7 (14.1)	38.6 (15.6)	39.1 (16.7)	0.78
CD4+ T_EFF_ (%)	3.2 (3.9)	3.3 (3.8)	4.7 (4.8)	0.093
CD4+ CD127+ (%)	92.4 (5.3)	90.7 (6.9)	88.4 (7.1)	< 0.001*
CD4+ CD28- (%)	7.1 (8.3)	9.0 (9.1)	7.9 (6.3)	0.12
CD4+ CD57+ (%)	4.2 (4.8)	5.3 (5.9)	5.1 (5.6)	0.096
CD8+ T_NAIVE_ (%)	22.9 (12.5)	19.9 (12.3)	18.5 (10.4)	0.02*
CD8+ T_EFF_ (%)	31.5 (17.5)	38.8 (19.4)	38.9 (19.6)	< 0.001*
CD8+ CD127+ (%)	55.1 (16.3)	46.9 (16.6)	47.8 (14.5)	< 0.001*
CD8+ CD28- (%)	45.2 (17.7)	54.8 (17.2)	53.8 (18.2)	< 0.001*
CD8+ CD57+ (%)	27.3 (17.4)	33.1 (20.5)	33.7 (18.0)	0.003*
Number CD68+	378.0 (146.7)	412.0 (162.8)	383.2 (159.4)	0.12
Monocyte 1 (%)	75.2 (8.9)	75.3 (9.9)	76.5 (9.1)	0.74
Monocyte 2 (%)	3.7 (1.7)	3.8 (2.4)	3.7 (1.9)	0.76
Monocyte 3 (%)	10.7 (4.7)	10.8 (7.0)	10.2 (5.7)	0.85

Immune cell profiles were compared between eGFR groups. *P* values were calculated using one-way ANOVA. For cell subsets, the percentage of the mother cell population was used for comparison instead of cell numbers. Values are expressed as means (SD). *: *p* value < 0.05. eGFR, estimated glomerular filtration rate derived from the CKD-EPI formula. Stage 3 CKD, eGFR between 30 and 60; stage 4 CKD, eGFR between 15 and 30; stage 5 CKD, eGFR less than 15. Monocyte 1: classical monocytes. Monocyte 2: intermediate monocytes. Monocyte 3: non-classical monocyte

### Independent association between CKD and immunosenescence

Next, we analyzed the independent effect of CKD on immunosenescence in age and gender-adjusted regression models (Table 3). There was a significant decrease in CD3^+^ and CD4^+^ T cells between patients with stage 4/5 CKD and patients with normal eGFR. In addition, stage 4/5 CKD patients exhibited lower percentages of CD4^+^CD127^+^ cells. Phenotypically, CD8^+^ subset changes were more pronounced than CD4+ cells. Among CD8+ T cells, the CD127^+^ subset was decreased and percentages of CD28^−^ as well as CD57^+^ cells were increased. There was a significant trend that as CKD stages increased, percentages of CD4^+^CD127^+^ cells, CD8+ T_NAIVE_ cells, and CD8^+^CD127^+^ cells decreased while percentages of effector CD8+ T_E_ cells, CD8^+^CD28^+^ cells, and CD8^+^CD57^+^ cells increased (Table 3). In the analysis, although age was adjusted in the multivariable-adjusted model, patients with normal eGFR were significantly younger than patients with CKD. To eliminate the concern of residual confounding effects of age, we further used the nearest neighbor matching to generate an age-matched sample of patients with normal eGFR > 60 ml/min (*N* = 138). The mean age of the age-matched sample was increased to 64.7 ± 9.32. We re-examined the relationship between CKD stages and immunophenotypes using this age-matched comparison group while still adjusting for age and sex. The results were similar to the main analysis (supplementary Table 2) suggesting that the relationship between CKD stages and immunophenotypes was independent of age.

**Table 3:** Immune cell number and phenotype by CKD stage in age and sex-adjusted regression models

	Stage 3 CKD versus eGFR ≥60		Stage 4/5 CKD versus eGFR ≥60		p for trend
	*ß*	*p value*	*ß*	*p value*	
Number CD3+	−18.09	0.68	− 163.13	0.021*	0.053
Number CD4+	−20.49	0.46	−110.87	0.013*	0.024*
Number CD8+	21.21	0.21	−37.09	0.17	0.73
CD4+ T_NAIVE_ (%)	1.75	0.29	2.19	0.41	0.23
CD4+ T_EFF_ (%)	−0.31	0.33	1.09	0.032*	0.29
CD4+ CD127+ (%)	−0.72	0.19	−3.21	< 0.001*	0.001*
CD4+ CD28- (%)	0.7	0.33	1.1	0.33	0.21
CD4+ CD57+ (%)	0.32	0.47	0.32	0.65	0.46
CD8+ T_NAIVE_ (%)	−2.59	0.045*	−3.24	0.12	0.024*
CD8+ T_EFF_ (%)	4.96	0.015*	4.9	0.14	0.015*
CD8+ CD127+ (%)	−4.66	0.009*	−3.59	0.21	0.019*
CD8+ CD28- (%)	6.04	0.002*	5.14	0.1	0.004*
CD8+ CD57+ (%)	3.45	0.041*	4.6	0.089	0.018*
Number CD68+	23.5	0.16	0.43	0.99	0.45
Monocyte 1 (%)	0.54	0.55	2.17	0.13	0.15
Monocyte 2 (%)	−0.19	0.29	−0.25	0.38	0.22
Monocyte 3 (%)	−0.78	0.13	−1.24	0.13	0.053

Age and sex-adjusted multivariable regression models and trend analyses to test the independent associations between immune profile and CKD stages. *: *P* value < 0.05. eGFR, estimated glomerular filtration rate derived from the CKD-EPI formula. Stage 3 CKD, eGFR between 30 and 60; stage 4 CKD, eGFR between 15 and 30; stage 5 CKD, eGFR less than 15. Monocyte 1: classical monocytes. Monocyte 2: intermediate monocytes. Monocyte 3: non-classical monocyte

These results indicate the existence of independent effects of CKD on T lymphocyte immunosenescence, and the effects emerge since stage 3 CKD and the phenotypical changes are more pronounced in the CD8^+^ T cell compartment.

### Neither the level of albuminuria nor that of serum glucose level significantly affects immunosenescence

Two recent reports found that level of senescent T cells predicts the future development of hyperglycemia [23, 24]. Thus, we analyzed the effects of hyperglycemia on immunosenescence. No effects were found for glycated hemoglobin and glucose level in the immune subsets tested (supplementary Table 3 and 4). In addition, no effects were found for duration of diabetes on immunosenescence (supplementary Table 5). We further analyzed the effects of specific types of oral hypoglycemia agent and insulin usage on immunosenescence (supplementary Table 6). However, none of the medications were associated with immunosenescence. Blood sugar level and medication used to control hyperglycemia were significantly different among three groups of patients. We further adjusted for blood glucose level and medication use in the regression models to understand if these factors modulate the effects of CKD on immunosenescence. The associations between CKD stages and lymphocyte immunosenescence remained consistent with the main analyses even after adjustment for glucose level (supplementary Table 7) as well as HbA1c and specific medication usage (data not shown).

Decline of renal function in diabetes is aggravated by the level of albuminuria. Nevertheless, when level of albuminuria was analyzed, only the CD8^+^CD28^−^ cells were found to increase with albuminuria with statistical significance (supplementary Table 8, *p* = 0.048). Since changes in other related cell subsets were not concomitantly significantly affected with albuminuria, we conclude that the effects of albuminuria on immunosenescence is much less prominent than the effects of the decrease in eGFR.

### Body mass index impacts immunosenescence

Finally, we investigated the effects of body mass index (BMI) on immunosenescence. BMI is closely related to thymic function in patients receiving kidney transplantation [25]. Enhanced T cell differentiation was also observed in children with excess BMI [26]. Whether BMI is associated with immunosenescence in diabetic adults is unknown. Because CKD has the potential to affect patients’ nutritional status and BMI, we investigated the effects of BMI in two regression models. As shown in Table 4, BMI positively associated with the increase in T cells with advanced differentiation but negatively associated with frequencies of naïve T cells, especially in the CD8^+^ compartment. While CD4^+^ T cells exhibit similar trend, most differences did not reach statistical significance. BMI did not impact on monocyte phenotypes in both models.

**Table 4:** Effects of BMI on immunosenescence in multivariable-adjusted regression models

	Model 1 Adjusted for Age, Sex		Model 2 Adjusted for Age, Sex, CKD status	
	*ß*	*p value*	*ß*	*p value*
CD4+ T_NAIVE_ (%)	−0.13	0.36	−0.19	0.20
CD4+ T_EFF_ (%)	0.0062	0.82	0.0081	0.77
CD4+ CD127+ (%)	−0.056	0.26	−0.038	0.44
CD4+ CD28- (%)	0.082	0.19	0.073	0.25
CD4+ CD57+ (%)	0.027	0.48	0.023	0.56
CD8+ T_NAIVE_ (%)	−0.20	0.083	−0.17	0.15
CD8+ T_EFF_ (%)	0.25	0.17	0.20	0.28
CD8+ CD127+ (%)	−0.25	0.12	−0.19	0.23
CD8+ CD28- (%)	0.37	0.033*	0.30	0.087
CD8+ CD57+ (%)	0.42	0.0049*	0.37	0.017*

Multivariable regression models to test the independent associations between immune profile and BMI. *: *P* value < 0.05. BMI: Body Mass Index. Monocyte 1: classical monocytes. Monocyte 2: intermediate monocytes. Monocyte 3: non-classical monocyte

## Discussion

The current study investigated the emergence and clinical correlation of immunosenescence in type 2 diabetic patients. To our knowledge, this is the largest cohort study focusing on immune changes in type 2 diabetic patients. Although we and others have proven that immunosenescence is an important feature of dialysis patients, this is the first evidence that immunosenescence occurs as early as stage 3 CKD during renal function decline and thus, suggests the mechanism of immunosenescence does not just occur after uremia or dialysis treatment but rather represents the chronic, collective physiological changes patients experience during the progression of CKD. In contrast, none of duration of diabetes, usage of specific oral hypoglycemic agent and nor the extent of albuminuria was significantly associated with immunosenescence.

A significant increase in T cell immunosenescence, as based on multiple markers (including naïve cell markers CD27/CD45RO, CD28, CD127 and CD57), is observed in type 2 diabetic patients with eGFR below 60 ml/min. At the same time, significant decreases in total T cell numbers as well as CD4+ and CD8+ T cell numbers also occur. Due to the decrease of T cell numbers and the increased percentages of cells with immunosenescent features, we determine that patients with CKD exhibit both qualitative as well as quantitative changes in their immunity which potentially leads to suboptimal responses to new infections and vaccinations.

Numerous past studies indicated that the risk of cardiovascular disease increases dramatically with declining renal function. It has been suggested that the renal failure-related risk is independent from traditional cardiovascular risk factors. Among the atypical cardiovascular risk factors associated with CKD, inflammation is probably the most consistent and strongest, and its role in this disease has been confirmed in many studies [27, 28]. Furthermore, inflammation is involved in almost all aging-related chronic diseases and thus, the concept of inflammaging is at the nexus of all the other mechanisms of aging [29]. In this regard, chronic inflammation is also closely related to immunosenescence. For example, the etiology of chronic inflammation is partially due to the secretory phenotype of senescent somatic and immune cells. Senescent immune cells spontaneously express proinflammatory cytokines without antigen stimulation [30]. In turn, an inflammatory milieu promotes the generation of senescent CD28^−^ T cells [31]. It is possible that inflammation and the accumulation of senescent immune cells collaboratively promote atherosclerosis. In this report, we demonstrate that immunosenescence parallels the elevated hs-CRP level seen in type 2 diabetic patients with renal function decline since stage 3 CKD, and thus implicates the potential collaborative contribution of inflammaging to the development of cardiovascular complications in type 2 diabetes.

Our results indicate that as CKD progress, progressive decrease in circulatory CD3+ T cells can be observed and the effect on cell number is more pronounced among CD4+ cells. This is bewildering because both CD4+ and CD8+ T cell numbers are decreased in ESRD [12]. In CKD, two studies [32, 33] found circulatory CD3+ T cell numbers are decreased and both showed the decrease is more pronounced in CD4+ T cells, consistent with ours. The reason behind such differences between CD4+ and CD8+ T cells remain unknown; although it may be due to the relative deficiency of IL-7 in CKD patients [32] and the stronger dependence of CD4+ T cells on IL-7 for homeostasis [34]. Another possibility is the preferential expansion of CMV-specific CD8+ T cells in patients with CKD or ESRD, as our recent study suggested that CMV might be less well-controlled in ESRD patients [13]. As a result, while quantitative changes are more pronounced in CD4^+^ T cells, qualitative changes might be more evident in the CD8+ compartment. Indeed, our recent report in ESRD patients [21] and others’ report in CKD patients [33] also showed the aging-related phenotypic changes are more pronounced in the CD8^+^ compartment than in CD4^+^ T cells. This is further demonstrated in the current study by the concordant decrease of CD8^+^ T_NAIVE_ and CD8^+^CD127^+^ cells along with the increase of CD8^+^T_E_, CD8^+^CD28^−^ and CD8^+^CD57^+^ cells in patients with CKD stage 3. Regretfully, we did not find that patients with stage 4/5 CKD have more CD8^+^CD28^−^ or CD28^+^CD57^+^ cells compared to stage 3 CKD patients; however, because the cohort contains relatively fewer CKD stage 4/5 patients, the comparison might be underpowered. We also found that CKD stage 3 patients simultaneously exhibited higher level of hs-CRP than stage 4/5 patients. The lack of dose effects of severity of renal failure on immunosenescence probably also reflects, the extent of inflammation and inflammaging changes in different levels of CKD.

Other factors also potentially modulate the effects of decreased renal function on enhanced immunosenescence. Mechanisms potentially contribute to immunosenescence during renal function decline includes decreased insulin-like growth factor 1 signaling pathway, decreased vitamin D level and accumulation of advanced glycation end products, because these factors are disturbed in CKD and found to be involved in the premature aging phenotype in certain organ systems. Among these, vitamin D in old individuals was found to correlate with some features of the immune system, mainly in the CD8^+^ T cell compartment [35]. The role of these factors in promoting immunosenescence requires further investigation.

Our study also identified the effects of BMI on advanced T cell differentiation, especially in the CD8^+^ compartment (Table 4). Such effect can still be observed after controlling for CKD status. Interestingly, a previous study found that plasma from obese individuals can induce the loss of CD28 expression in PBMCs from healthy individuals with normal BMI. Other features of senescence, including decreased expression of γ-H2AX and p53, can also be observed [36]. Importantly, the effects of obesity on immunosenescence may be reversible, as exercise has been shown to improve immunosenescence [37].

In our study, aging-related subset distribution changes could only be observed in the T lymphocyte compartment, while the monocyte subset distribution and number remained mostly unchanged with renal function decline. Studies of the impact of aging on the function and homeostasis of human monocytes are limited in number and have yielded conflicting results. While some studies had shown that aging influences monocyte subset distribution [38, 39], others found that aging does not have an effect on the surface phenotype of monocytes in non-stimulated conditions [40]. Our current study indicates that in type 2 diabetic patients, aging is associated with the increase of proinflammatory intermediate and non-classical monocyte subsets. On the other hand, we did not observe significant effects of renal function on the monocyte compartment in type 2 diabetic patients. A previous study indicated that monocyte functionality in response to stimulation of pattern recognition receptors changes during aging [41]; however, we did not measure the response of monocytes in vitro in this study. Thus, monocytes might undergo more significant changes after dialysis treatment since intermediate CD14^++^CD16^+^ monocytes and non-classical monocytes are both increased in hemodialysis patients [21].

Our study has several additional limitations. First, while the seroprevalence of cytomegalovirus (CMV) in the study group is not known, the overall CMV seroprevalence in Taiwan is known to be very high. In addition, based on our recent study, the seroprevalence of CMV is almost 99% in this age group in Taiwan [42]. It is possible that effects of renal failure can only be observed in patients infected with CMV, as evidenced in a recent publication [33] and analyzed in our recent review [13]. Secondly, because this study only includes diabetic patients, our findings may not be generalizable to non-diabetic patients with CKD. Finally, longitudinal follow-up will be necessary to determine if the progression of immunosenescence precedes or parallels the deterioration of renal function.

## Conclusion

Type 2 diabetic patients exhibit significant aging-related immune changes in the T cell compartment in parallel with enhanced systemic inflammation as eGFR declines to less than 60 ml/min (CKD stage 3). Immunosenescence positively associates with higher BMI. This finding has important implications for further intervention studies and suggests that, in type 2 diabetic patients, therapeutic measures aimed at modulating immunosenescence and inflammation should be initiated prior to the development of stage 3 CKD.

## Supplementary information

**Additional file 1:** **Supplementary Figure 1** Representative flow cytometry staining of immunophenotypin. **Supplementary Table 1** Correlations between Immune cell number and phenotype with age. **Supplementary Table 2** Immune cell number and phenotype by CKD stage in age and sex-adjusted regression models in age-matched samples **Supplementary Table 3** Effects of HbA1c on immune cell subsets in multivariable-adjusted regression models. **Supplementary Table 4** Effects of glucose level on immune cell subsets in multivariable-adjusted regression model. **Supplementary Table 5** Effects of duration of diabetes on immune cell subsets in multivariable-adjusted regression models. **Supplementary Table 6** Effects of specific glucose-lowering medication usage on immune cell subsets in multivariable-adjusted regression models. **Supplementary Table 7** Effects of CKD on immune cell subsets in age, sex and glucose level-adjusted regression models. **Supplementary Table 8** Immune cell number and phenotype comparisons by albuminuria levels.

## Data Availability

All relevant data are within the manuscript and its supporting Information files.

## Abbreviations

ESRD: End-stage renal disease
CKD: Chronic kidney disease
CVD: Cardiovascular disease
eGFR: Estimated glomerular filtration rate
BMI: Body mass index
CMV: Cytomegalovirus
hs-CRP: High-sensitivity C-reactive protein
