# Supplementary material for: Emergence of T cell immunosenescence in diabetic chronic kidney disease
Source: Immun Ageing. 2020 Oct 20;17:31. doi: 10.1186/s12979-020-00200-1 (PMC7574244; doi:10.1186/s12979-020-00200-1)
Supplement: Supplementary file 1 — Additional file 1: Supplementary Figure 1 Representative flow cytometry staining of immunophenotypin. Supplementary Table 1 Correlations between Immune cell number and phenotype with age. Supplementary Table 2 Immune cell number and phenotype by CKD stage in age and sex-adjusted regression models in age-matched samples Supplementary Table 3 Effects of HbA1c on immune cell subsets in multivariable-adjusted regression models. Supplementary Table 4 Effects of glucose level on immune cell subsets in multivariable-adjusted regression model. Supplementary Table 5 Effects of duration of diabetes on immune cell subsets in multivariable-adjusted regression models. Supplementary Table 6 Effects of specific glucose-lowering medication usage on immune cell subsets in multivariable-adjusted regression models. Supplementary Table 7 Effects of CKD on immune cell subsets in age, sex and glucose level-adjusted regression models. Supplementary Table 8 Immune cell number and phenotype comparisons by albuminuria levels. [file 12979_2020_200_MOESM1_ESM.docx]

**Supporting Information**

**Supplementary Figure 1**

**Representative flow cytometry staining of immunophenotyping**

**
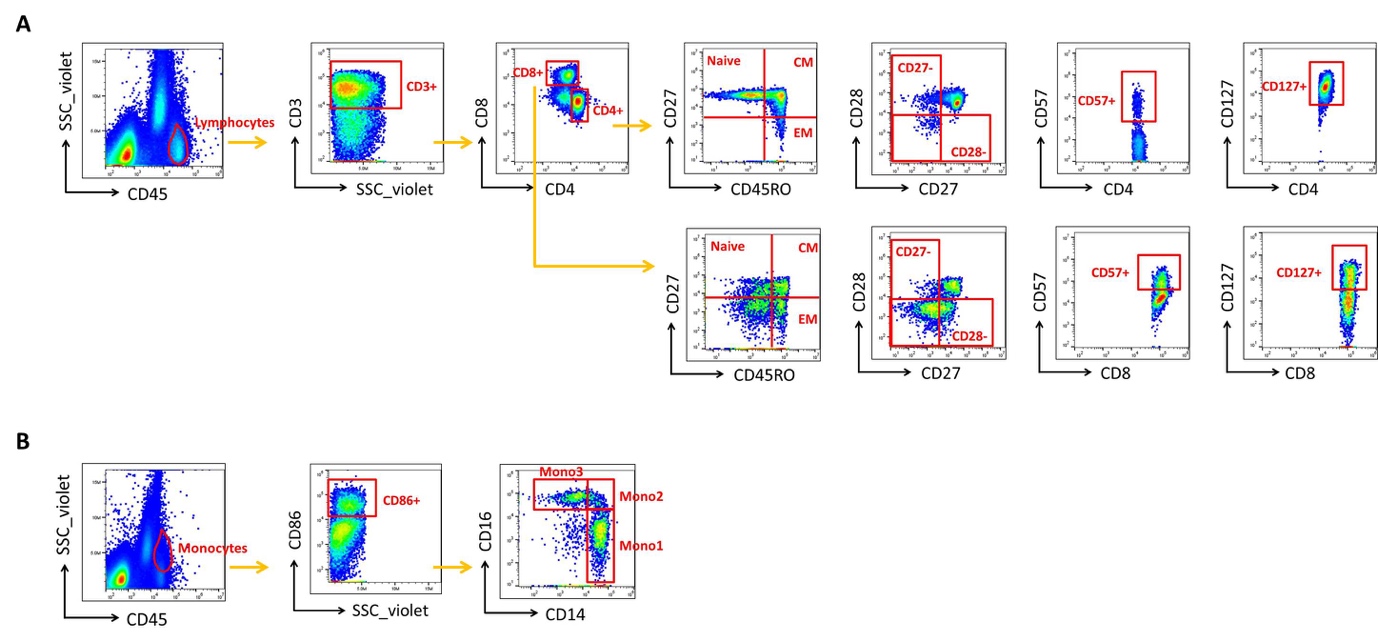
**

Representative flow cytometry plot and gating strategy as described in the Methods section. CM: central memory T cell. EM: effector memory T cell. CD45RO-CD27-: terminal effector T cell. Mono1: classical monocyte; Mono2: intermediate monocyte; Mono3: non-classical monocyte.

**Supplementary Table 1**

**Correlations between Immune cell number and phenotype with age**

| T cells | R^2^ | p value |
| --- | --- | --- |
| CD3+ number | -0.153 | 0.0004** |
| CD4+ number | -0.118 | 0.0067* |
| CD8+ number | -0.117 | 0.0074** |
| CD4+ T_NAIVE_ % | -0.1 | 0.021* |
| CD4+ T_CM_ % | -0.072 | 0.1 |
| CD4+ T_EM_ % | 0.208 | <0.001** |
| CD4+ T_E_ % | 0.041 | 0.35 |
| CD4+ CD127+ % | -0.115 | 0.0082* |
| CD4+ CD27- % | 0.15 | 0.006* |
| CD4+ CD28- % | 0.187 | <0.001** |
| CD4+ 57+ % | 0.149 | 0.0006** |
| CD8+ T_NAIVE_ % | -0.116 | 0.0077* |
| CD8+ T_CM_ % | -0.275 | <0.0001** |
| CD8+ T_EM_ % | 0.138 | 0.0015* |
| CD8+ T_E_ % | 0.209 | <0.0001** |
| CD8+ CD127+ % | -0.315 | <0.0001** |
| CD8+ CD27- % | 0.178 | <0.0001** |
| CD8+ CD28- % | 0.31 | <0.0001** |
| CD8+ 57+ % | 0.146 | 0.0008* |

| Monocytes | R^2^ | p value |
| --- | --- | --- |
| CD68+ number | 0.058 | 0.18 |
| CD14++CD16-  Classical monocyte % | -0.017 | 0.69 |
| CD14++CD16+  Intermediate monocyte % | 0.124 | 0.0047* |
| CD14+ CD16++  Non-classical monocyte % | 0.091 | 0.038* |

Pearson’s correlation analyses between age and immune cell parameters in all the participants. For simplicity and to avoid biases caused by the progressive decrease in total T cell number during aging, absolute immune cell numbers only were used for CD3+, CD4+, CD8+, and CD68 total monocytes. All other cellular subsets were analyzed by subset frequency of the mother cell population. *: P value < 0.05; **: p value < 0.001.

**Supplementary Table 2**

**Immune cell number and phenotype by CKD stage in age and sex-adjusted regression models in age-matched samples**

|  | | eGFR ≥ 60  versus  Stage 3 CKD | | eGFR ≥ 60  versus  Stage 4/5 CKD | | p for trend |
| --- | --- | --- | --- | --- | --- | --- |
|  | *ß* | | *p value* | *ß* | *p value* |  |
| Number CD3+ | 15.11 | | 0.76 | -125.02 | 0.097 | 0.25 |
| Number CD4+ | -6.85 | | 0.83 | -97.11 | 0.039* | 0.091 |
| Number CD8+ | 36.6 | | 0.06 | -19.26 | 0.50 | 0.76 |
| CD4+ T_NAIVE_ (%) | 2.06 | | 0.28 | 1.88 | 0.51 | 0.32 |
| CD4+ T_EFF_ (%) | -0.46 | | 0.22 | 0.93 | 0.1 | 0.47 |
| CD4+ CD127+ (%) | -0.84 | | 0.20 | -3.43 | 0.0005* | 0.0012 |
| CD4+ CD28- (%) | 0.88 | | 0.27 | 1.26 | 0.29 | 0.19 |
| CD4+ CD57+ (%) | 0.2 | | 0.70 | 0.23 | 0.77 | 0.69 |
| CD8+ T_NAIVE_ (%) | -2.57 | | 0.07 | -2.91 | 0.17 | 0.067 |
| CD8+ T_EFF_ (%) | 5.83 | | 0.017* | 5.63 | 0.12 | 0.026 |
| CD8+ CD127+ (%) | -5.6 | | 0.007* | -4.05 | 0.19 | 0.028 |
| CD8+ CD28- (%) | 6.56 | | 0.0035* | 5.25 | 0.12 | 0.013 |
| CD8+ CD57+ (%) | 3.75 | | 0.053 | 4.96 | 0.087 | 0.030 |
| Number CD68+ | 20.49 | | 0.29 | -5.1 | 0.86 | 0.75 |
| Monocyte 1 (%) | 0.71 | | 0.48 | 2.16 | 0.15 | 0.16 |
| Monocyte 2 (%) | -0.26 | | 0.21 | -0.35 | 0.26 | 0.15 |
| Monocyte 3 (%) | -0.81 | | 0.16 | -1.31 | 0.13 | 0.072 |

The nearest neighbor matching was used to generate an age-matched sample of patients with normal eGFR > 60 ml/min (N=138). The mean age of the age-matched samples was 64.7 ± 9.32. We re-examined the relationship between CKD stages and immunophenotypes using this age-matched comparison group while still adjusting for age and sex. The results were similar to the main analysis confirming that the relationship between CKD stages and immunophenotypes was independent of age effect on immunophenotypes.

**Supplementary Table 3**

**Effects of HbA1c on immune cell subsets in multivariable-adjusted regression models**

|  | Model 1  Adjusted for Age, Sex, CKD status | | Model 2  Adjusted for Age, Sex, eGFR | |
| --- | --- | --- | --- | --- |
|  | *ß* | *p value* | *ß* | *p value* |
| Number CD3+ | 39.06 | 0.017* | 40.16 | 0.014* |
| Number CD4+ | 22.88 | 0.026* | 23.35 | 0.023* |
| Number CD8+ | 7.52 | 0.23 | 8.32 | 0.18 |
| CD4+ T_NAIVE_ (%) | 0.47 | 0.44 | 0.48 | 0.43 |
| CD4+ T_EFF_ (%) | -0.05 | 0.66 | -0.07 | 0.54 |
| CD4+ CD127+ (%) | 0.15 | 0.45 | 0.18 | 0.38 |
| CD4+ CD28- (%) | -0.12 | 0.66 | -0.12 | 0.65 |
| CD4+ CD57+ (%) | -0.16 | 0.33 | -0.16 | 0.32 |
| CD8+ T_NAIVE_ (%) | -0.26 | 0.58 | -0.29 | 0.54 |
| CD8+ T_EFF_ (%) | 0.43 | 0.57 | 0.48 | 0.53 |
| CD8+ CD127+ (%) | 0.83 | 0.21 | 0.77 | 0.24 |
| CD8+ CD28- (%) | -0.84 | 0.24 | -0.77 | 0.29 |
| CD8+ CD57+ (%) | -0.6 | 0.33 | -0.57 | 0.36 |
| Number CD68+ | 4.72 | 0.45 | 4.81 | 0.44 |
| Monocyte 1 (%) | 0.56 | 0.1 | 0.53 | 0.11 |
| Monocyte 2 (%) | -0.03 | 0.66 | -0.03 | 0.68 |
| Monocyte 3 (%) | -0.27 | 0.17 | -0.26 | 0.17 |

Multivariable regression models to test the independent associations between immune profile and HbA1C. *: P value < 0.05. eGFR, estimated glomerular filtration rate derived from the CKD-EPI formula. HbA1c: Glycated hemoglobin. Monocyte 1: classical monocytes. Monocyte 2: intermediate monocytes. Monocyte 3: non-classical monocyte.

**Supplementary Table 4**

**Effects of fasting glucose level on immune cell subsets in multivariable-adjusted regression models**

|  | Model 1  Adjusted for Age, Sex, CKD status | | Model 2  Adjusted for Age, Sex, eGFR | |
| --- | --- | --- | --- | --- |
|  | *ß* | *p value* | *ß* | *p value* |
| Number CD3+ | 0.58 | 0.14 | 0.63 | 0.11 |
| Number CD4+ | 0.25 | 0.30 | 0.28 | 0.25 |
| Number CD8+ | 0.19 | 0.20 | 0.23 | 0.13 |
| CD4+ T_NAIVE_ (%) | -0.0003 | 0.98 | 0.0003 | 0.98 |
| CD4+ T_EFF_ (%) | -0.0022 | 0.42 | -0.003 | 0.28 |
| CD4+ CD127+ (%) | -0.0004 | 0.94 | 0.0004 | 0.94 |
| CD4+ CD28- (%) | -0.0041 | 0.51 | -0.0038 | 0.53 |
| CD4+ CD57+ (%) | -0.0051 | 0.19 | -0.0049 | 0.21 |
| CD8+ T_NAIVE_ (%) | -0.0026 | 0.82 | -0.0035 | 0.76 |
| CD8+ T_EFF_ (%) | -0.0075 | 0.68 | -0.005 | 0.78 |
| CD8+ CD127+ (%) | 0.016 | 0.32 | 0.013 | 0.41 |
| CD8+ CD28- (%) | -0.015 | 0.39 | -0.012 | 0.51 |
| CD8+ CD57+ (%) | -0.014 | 0.36 | -0.012 | 0.42 |
| Number CD68+ | -0.088 | 0.56 | -0.063 | 0.67 |
| Monocyte 1 (%) | 0.017 | 0.04 | 0.016 | 0.04 |
| Monocyte 2 (%) | -0.002 | 0.21 | -0.0021 | 0.20 |
| Monocyte 3 (%) | -0.0049 | 0.29 | -0.005 | 0.28 |

Multivariable regression models to test the independent associations between immune profile and fasting glucose level. *: P value < 0.05. eGFR, estimated glomerular filtration rate derived from the CKD-EPI formula. HbA1c: Glycated hemoglobin. Monocyte 1: classical monocytes. Monocyte 2: intermediate monocytes. Monocyte 3: non-classical monocyte.

**Supplementary Table 5**

**Effects of duration of diabetes on immune cell subsets in multivariable-adjusted regression models**

|  | Model 1  Adjusted for Age, Sex | | Model 2  Adjusted for Age, Sex, CKD status | |
| --- | --- | --- | --- | --- |
|  | *ß* | *p value* | *ß* | *p value* |
| Number CD3+ | -1.35 | 0.64 | -0.57 | 0.85 |
| Number CD4+ | -1.88 | 0.31 | -1.35 | 0.47 |
| Number CD8+ | -0.037 | 0.97 | -0.085 | 0.94 |
| CD4+ T_NAIVE_ (%) | 0.1 | 0.36 | 0.085 | 0.44 |
| CD4+ T_EFF_ (%) | 0.0066 | 0.75 | 0.0062 | 0.76 |
| CD4+ CD127+ (%) | 0.0054 | 0.88 | 0.018 | 0.60 |
| CD4+ CD28- (%) | 0.031 | 0.49 | 0.03 | 0.51 |
| CD4+ CD57+ (%) | -0.0037 | 0.90 | -0.0042 | 0.89 |
| CD8+ T_NAIVE_ (%) | -0.065 | 0.45 | -0.037 | 0.67 |
| CD8+ T_EFF_ (%) | -0.015 | 0.91 | -0.071 | 0.60 |
| CD8+ CD127+ (%) | 0.11 | 0.36 | 0.16 | 0.18 |
| CD8+ CD28- (%) | 0.028 | 0.83 | -0.037 | 0.77 |
| CD8+ CD57+ (%) | 0.033 | 0.76 | -0.0016 | 0.99 |
| Number CD68+ | -0.49 | 0.66 | -0.6 | 0.59 |
| Monocyte 1 (%) | 0.047 | 0.44 | 0.035 | 0.56 |
| Monocyte 2 (%) | -0.022 | 0.06 | -0.021 | 0.08 |
| Monocyte 3 (%) | -0.062 | 0.07 | -0.053 | 0.12 |

Multivariable regression models to test the independent associations between immune profile and duration of diabetes. *: P value < 0.05. Monocyte 1: classical monocytes. Monocyte 2: intermediate monocytes. Monocyte 3: non-classical monocyte.

**Supplementary Table 6**

**Effects of specific glucose-lowering medication usage on immune cell subsets in multivariable-adjusted regression models**

|  |  | Model 1  Adjusted for Age, Sex | | Model 2  Adjusted for Age, Sex, CKD status | |
| --- | --- | --- | --- | --- | --- |
|  |  | *ß* | *p value* | *ß* | *p value* |
| Insulin | CD4+ T_NAIVE_ (%) | 12.4 | 0.73 | 37.99 | 0.32 |
|  | CD8+ T_NAIVE_ (%) | 0.85 | 0.53 | 0.68 | 0.63 |
|  | CD8+ T_EFF_ (%) | -0.76 | 0.48 | -0.19 | 0.86 |
|  | CD8+ CD127+ (%) | -0.09 | 0.96 | -1.23 | 0.48 |
|  | CD8+ CD28- (%) | 0.8 | 0.59 | 1.87 | 0.22 |
| Sulfonylurea | CD4+ T_NAIVE_ (%) | -0.15 | 0.91 | -0.14 | 0.91 |
|  | CD8+ T_NAIVE_ (%) | 0.71 | 0.48 | 0.56 | 0.58 |
|  | CD8+ T_EFF_ (%) | -1.13 | 0.48 | -0.88 | 0.58 |
|  | CD8+ CD127+ (%) | 2.39 | 0.09 | 2.20 | 0.11 |
|  | CD8+ CD28- (%) | -1.63 | 0.29 | -1.39 | 0.36 |
| Metformin | CD4+ T_NAIVE_ (%) | -1.15 | 0.43 | -0.69 | 0.68 |
|  | CD8+ T_NAIVE_ (%) | 0.99 | 0.38 | -0.38 | 0.77 |
|  | CD8+ T_EFF_ (%) | -3.53 | 0.05 | -1.74 | 0.4 |
|  | CD8+ CD127+ (%) | 3.51 | 0.03* | 2.22 | 0.22 |
|  | CD8+ CD28- (%) | -4.08 | 0.02* | -2.16 | 0.27 |
| DPP4i | CD4+ T_NAIVE_ (%) | -0.89 | 0.49 | -1.04 | 0.42 |
|  | CD8+ T_NAIVE_ (%) | -0.37 | 0.71 | -0.31 | 0.76 |
|  | CD8+ T_EFF_ (%) | 1.92 | 0.23 | 1.8 | 0.25 |
|  | CD8+ CD127+ (%) | 0.35 | 0.8 | 0.46 | 0.74 |
|  | CD8+ CD28- (%) | 1.04 | 0.49 | 0.88 | 0.56 |
| SGLT2i | CD4+ T_NAIVE_ (%) | -111.76 | 0.07 | -127.93 | 0.04* |
|  | CD8+ T_NAIVE_ (%) | -4.45 | 0.05 | -4.25 | 0.07 |
|  | CD8+ T_EFF_ (%) | -2.09 | 0.24 | -3.08 | 0.09 |
|  | CD8+ CD127+ (%) | -1.68 | 0.55 | -0.12 | 0.97 |
|  | CD8+ CD28- (%) | -2.07 | 0.4 | -3.34 | 0.18 |

Multivariable regression models to test the independent associations between selected immune subset and glucose-lowering medications. DPP4i: Dipeptidyl peptidase-4 inhibitor. SGLT2i: sodium-glucose cotransporter 2 inhibitor. *: P value < 0.05.

**Supplementary Table 7**

**Immune cell number and phenotype by CKD stage in age, sex and glucose level-adjusted regression models**

|  | | eGFR ≥ 60  versus  Stage 3 CKD | eGFR ≥ 60  versus  Stage 4/5 CKD | | p for trend |
| --- | --- | --- | --- | --- | --- |
|  | *ß* | *p value* | *ß* | *p value* |  |
| Number CD3+ | -23.9 | 0.59 | -156.07 | 0.028* | 0.053 |
| Number CD4+ | -23.06 | 0.41 | -107.81 | 0.016* | 0.024* |
| Number CD8+ | 19.36 | 0.25 | -34.73 | 0.20 | 0.73 |
| CD4+ T_NAIVE_ (%) | 1.75 | 0.29 | 2.19 | 0.41 | 0.23 |
| CD4+ T_EFF_ (%) | -0.29 | 0.37 | 1.06 | 0.038* | 0.29 |
| CD4+ CD127+ (%) | -0.72 | 0.19 | -3.21 | 0.0003* | 0.0007* |
| CD4+ CD28- (%) | 0.74 | 0.29 | 1.05 | 0.35 | 0.21 |
| CD4+ CD57+ (%) | 0.38 | 0.40 | 0.26 | 0.71 | 0.46 |
| CD8+ T_NAIVE_ (%) | -2.56 | 0.048* | -3.27 | 0.11 | 0.024* |
| CD8+ T_EFF_ (%) | 5.04 | 0.014* | 4.81 | 0.14 | 0.015* |
| CD8+ CD127+ (%) | -4.82 | 0.0073* | -3.4 | 0.24 | 0.019* |
| CD8+ CD28- (%) | 6.19 | 0.0017* | 4.95 | 0.11 | 0.004* |
| CD8+ CD57+ (%) | 3.61 | 0.034* | 4.44 | 0.10 | 0.018* |
| Number CD68+ | 24.52 | 0.14 | -0.51 | 0.98 | 0.45 |
| Monocyte 1 (%) | 0.36 | 0.69 | 2.35 | 0.10 | 0.15 |
| Monocyte 2 (%) | -0.17 | 0.35 | -0.27 | 0.34 | 0.23 |
| Monocyte 3 (%) | -0.73 | 0.16 | -1.29 | 0.12 | 0.053 |

Age and sex-adjusted multivariable regression models and trend analyses to test the independent associations between immune profile and CKD stages. *: P value < 0.05. eGFR, estimated glomerular filtration rate derived from the CKD-EPI formula. Stage 3 CKD, eGFR between 30 and 60; stage 4 CKD, eGFR between 15 and 30; stage 5 CKD, eGFR less than 15. Monocyte 1: classical monocytes. Monocyte 2: intermediate monocytes. Monocyte 3: non-classical monocyte.

**Supplementary Table 8**

**Immune cell number and phenotype comparisons by albuminuria**

|  | No albuminuria  ACR<30  (n=483) | Microalbuminuria  300≥ACR≥30  (n=199) | Macroalbuminuria  ACR≥300  (n=82) | p value |
| --- | --- | --- | --- | --- |
| Number CD3+ | 987.2 (333.5) | 1003.5 (375.9) | 979.6 (382.0) | 0.89 |
| Number CD4+ | 585.0 (221.7) | 597.4 (252.5) | 555.2 (238.1) | 0.56 |
| Number CD8+ | 271.1 (142.2) | 277.9 (141.8) | 301.7 (145.8) | 0.38 |
| CD4+ T_NAIVE_ (%) | 41.1 (10.4) | 43.6 (10.9) | 40.8 (9.8) | 0.059 |
| CD4+ T_EFF_ (%) | 3.2 (3.5) | 3.5 (4.7) | 3.0 (3.1) | 0.55 |
| CD4+ CD127+ (%) | 92.2 (5.6) | 91.7 (5.7) | 92.1 (5.0) | 0.67 |
| CD4+ CD28- (%) | 7.3 (8.6) | 7.4 (8.3) | 6.7 (5.2) | 0.86 |
| CD4+ CD57+ (%) | 4.3 (4.7) | 4.5 (5.6) | 3.9 (3.7) | 0.71 |
| CD8+ T_NAIVE_ (%) | 22.7 (12.4) | 22.0 (13.8) | 20.6 (11.3) | 0.54 |
| CD8+ T_EFF_ (%) | 31.7 (18.4) | 35.0 (18.3) | 37.7 (17.8) | 0.050 |
| CD8+ CD127+ (%) | 54.1 (17.1) | 52.6 (16.8) | 48.1 (13.7) | 0.063 |
| CD8+ CD28- (%) | 46.2 (18.5) | 48.3 (18.0) | 52.9 (15.3) | 0.048* |
| CD8+ CD57+ (%) | 28.5 (18.9) | 28.4 (16.1) | 31.5 (21.0) | 0.56 |
| Number CD68+ | 378.3 (141.3) | 392.4 (139.4) | 421.8 (159.3) | 0.13 |
| Monocyte 1 (%) | 75.4 (8.3) | 75.3 (8.7) | 74.6 (12.5) | 0.86 |
| Monocyte 2 (%) | 3.7 (1.6) | 3.6 (1.5) | 3.8 (2.5) | 0.75 |
| Monocyte 3 (%) | 10.8 (4.7) | 10.7 (4.3) | 10.7 (9.1) | 0.99 |

Immune cell profiles were compared between albuminuria groups. Continuous variables are described as mean (SD) for parametric variables and median (interquartile range) for nonparametric variables unless otherwise indicated. The P values were calculated using one-way ANOVA. For cell subsets, the percentage of the mother cell population was used for comparison instead of cell numbers. Values are expressed as means (SD). *: p value < 0.05. Monocyte 1: classical monocytes. Monocyte 2: intermediate monocytes. Monocyte 3: non-classical monocyte.
